# Correction: Avetissian et al. A Novel Piezoelectric Energy Harvester for Earcanal Dynamic Motion Exploitation Using a Bistable Resonator Cycled by Coupled Hydraulic Valves Made of Collapsed Flexible Tubes. *Micromachines* 2024, *15*, 415

**DOI:** 10.3390/mi15111291

**Published:** 2024-10-23

**Authors:** Tigran Avetissian, Fabien Formosa, Adrien Badel, Aidin Delnavaz, Jérémie Voix

**Affiliations:** 1 Université du Québec-École de Technologie Supérieure, Montréal, QC H3C 1K3, Canada; cc-aidin.delnavaz@etsmt.ca (A.D.); jeremie.voix@etsmtl.ca (J.V.); 2 Laboratoire SYMME, Université Savoie Mont Blanc, 74940 Annecy, France; fabien.formosa@univ-smb.fr (F.F.); adrien.badel@univ-smb.fr (A.B.)

## 1. Correction in Affiliation

In the original publication [1], the affiliation Université Savoie Mont Blanc-Laboratoire SYMME should be Laboratoire SYMME, Université Savoie Mont Blanc. In the original publication, corresponding author was tigran.avetissian@etsmtl.ca, while it should be tavetissian@critias.ca.

## 2. Error in Figures 3 and 4

In the original publication, there were mistakes in Figures 3 and 4 as published. The figures appeared in the wrong order because the LaTeX file did not match with the correct “figures” folder. Figure 3 and Figure 4 should appear as follows, in the specified order.

## 3. Error in Figure 11

In the original publication [1], there was a mistake in Figure 11 as published. The version of the figure was obsolete. It was updated after the first round of the review process. The updated version of Figure 11 appears below.

The authors state that the scientific conclusions are unaffected. This correction was approved by the Academic Editor. The original publication has also been updated.

## Figures and Tables

**Figure 3:**
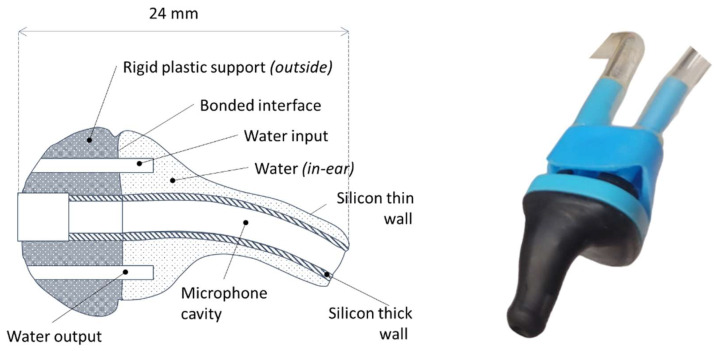
Earplug presentation. (**a**) Earplug schema. (**b**) Earplug picture.

**Figure 4:**
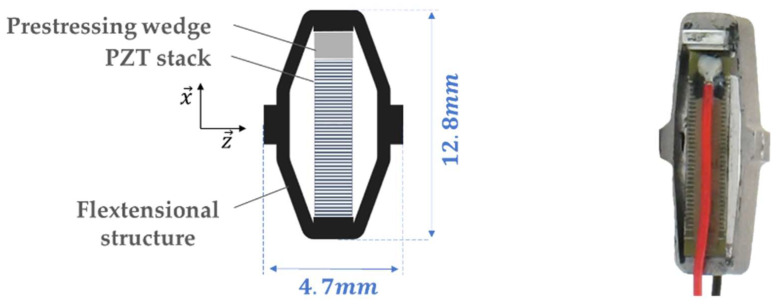
Amplified piezoelectric generator (APG). (**a**) APG detailed schema. (**b**) APG picture.

**Figure 11:**
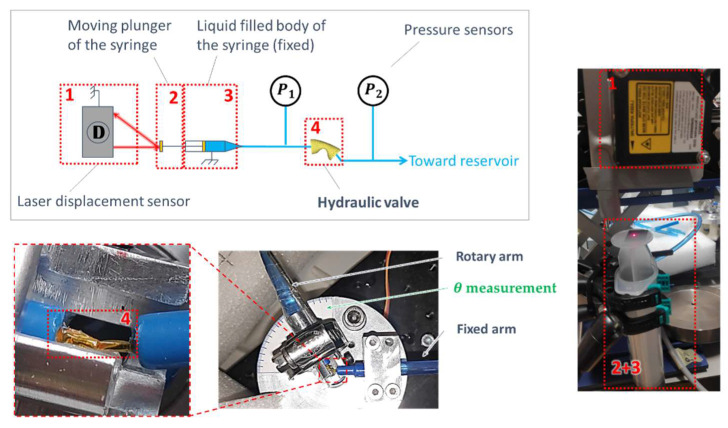
Schematic view and pictures of the hydraulic characterization test bench for the HVs.
